# Evaluation of menstrual irregularities after COVID-19 vaccination: Results of the MECOVAC survey

**DOI:** 10.1515/med-2022-0452

**Published:** 2022-03-09

**Authors:** Antonio Simone Laganà, Giovanni Veronesi, Fabio Ghezzi, Marco Mario Ferrario, Antonella Cromi, Mariano Bizzarri, Simone Garzon, Marco Cosentino

**Affiliations:** Department of Obstetrics and Gynecology, “Filippo Del Ponte” Hospital, University of Insubria, 21100, Varese, Italy; Department of Medicine and Surgery, School of Medicine, Research Center in Epidemiology and Preventive Medicine, University of Insubria, 21100, Varese, Italy; Occupational Medicine Unit, University Hospital of Varese, 21100, Varese, Italy; Department of Experimental Medicine, Systems Biology Group Lab, University La Sapienza, 00161, Rome, Italy; Department of Obstetrics and Gynaecology, AOUI Verona, University of Verona, 37126, Verona, Italy; Center for Research in Medical Pharmacology, University of Insubria, 21100, Varese, Italy

**Keywords:** COVID-19, vaccine, menstrual irregularities, abnormal uterine bleeding, adverse effect

## Abstract

We investigated menstrual irregularities after the first and second doses of the COVID-19 vaccine. Women answered a customised online questionnaire (ClinicalTrial.gov ID: NCT05083065) aimed to assess the vaccine type, the phase of the menstrual cycle during which the vaccine was administered, the occurrence of menstrual irregularities after the first and second doses, and how long this effect lasted. We excluded women with gynaecological and non-gynaecological diseases, undergoing hormonal and non-hormonal treatments, in perimenopause or menopause, as well as those who had irregular menstrual cycles in the last 12 months before vaccine administration. According to our data analysis, approximately 50–60% of reproductive-age women who received the first dose of the COVID-19 vaccine reported menstrual cycle irregularities, regardless of the type of administered vaccine. The occurrence of menstrual irregularities seems to be slightly higher (60–70%) after the second dose. Menstrual irregularities after both the first and second doses of the vaccine were found to self-resolve in approximately half the cases within two months. Based on these results, we suggest to consider these elements during the counselling of women who receive the COVID-19 vaccine, letting them know about the potential occurrence of temporary and self-limiting menstrual cycle irregularities in the subsequent month(s).

## 1 Introduction

Italy was one of the first countries where the COVID-19 pandemic started [1], after the initial spread in Wuhan, China [2]. Lombardy and Veneto, as well as other northern areas of Italy, had the highest morbidity and mortality rates since they were suddenly hit by the new virus, whose biological and clinical effects were yet unknown [3]. Among the potential strategies to counteract the pandemic, since January 2021, Italy started a campaign to increase the vaccination rate of healthcare providers, fragile patients, and general population as much as possible [4]. Four different vaccines were used: Comirnaty (Pfizer-BioNTech), Spikevax (Moderna), and Vaxzevria (AstraZeneca), which could be administered for the first and second doses, and Janssen (Johnson & Johnson) in a single administration. In addition, since 15 October 2021, the Italian government required to have a valid European Digital COVID Certificate (EDCC) for all productive activities and to work [5], and this further increased the vaccination rate among the general population, similar to other countries [6]. Notably, data from the EudraVigilance European database (23 June 2021) show in young adult (18–64 years old) and older (≥65 years old) recipients a higher number of severe adverse events caused by venous blood clots, haemorrhage, thromboembolic disease, and arterial events, including myocardial infarction and stroke, with the use of virus-based COVID-19 vaccines than other types of vaccines [7].

From our local perspective, we started to notice an increased number of outpatient visits for menstrual irregularities and abnormal uterine bleeding in women who received the COVID-19 vaccine. Interestingly, although the UK’s Medicines and Healthcare products Regulatory Agency (MHRA) stated that the evaluation of yellow card surveillance reports does not support a link between changes in menstrual periods and COVID-19 vaccines, a potential link has been recently suggested and solicited further investigation [8]. In this scenario, the National Institutes of Health (NIH) recently awarded one-year supplemental grants, totalling $1.67 million, to five institutions for exploring potential links between COVID-19 vaccination and menstrual changes [9], since some women have reported experiencing irregular or missing menstrual periods, bleeding that is heavier than usual, and other menstrual changes after receiving COVID-19 vaccines. Interestingly, in a recent study on women of childbearing age, approximately 25% of patients infected with COVID-19 experienced menstrual disruption [10]. In addition, women infected with COVID-19 were found to have a decrease in menstruation duration compared with their own pre-pandemic data: in particular, Demir et al. found a positive correlation between the stress and anxiety associated with COVID-19 and menstrual cycle dysregulations [11].

Based on these elements, we decided to investigate the occurrence of menstrual irregularities and abnormal uterine bleeding after the first and second doses of the COVID-19 vaccine, correlate these cases to the type of vaccine administered, and evaluate how long this effect lasted.

## 2 Methods

### 2.1 Setting, data sources, and study population

We designed a customised pilot questionnaire (menstruation after COVID vaccine, MECOVAC) consisting of 26 multiple-choice questions. The first 13 items aimed to assess age, body mass index, concurrent gynaecological and non-gynaecological disorders, use hormonal and non-hormonal pharmacological treatments, number of previous pregnancies and abortions, reproductive or (peri)menopausal status, and type of COVID-19 vaccine used for the first and second doses (single in case of Janssen by Johnson & Johnson). The following three questions (items 14–16) assessed the frequency, length, and quantity of the menstrual cycles before the first dose of the COVID-19 vaccine (single in the case of Janssen by Johnson & Johnson). The following five questions (items 17–21) assessed the phase of the menstrual cycle in which the first dose of the COVID-19 vaccine (single in the case of Janssen by Johnson & Johnson) was administered, the frequency, length, and quantity of the menstrual cycle after the administration, and how long this effect lasted in case of menstrual cycle irregularities and/or abnormal uterine bleeding. The last five questions (items 22–26) assessed the phase of the menstrual cycle in which the second dose of the COVID-19 vaccine (not applicable in case of the single dose of Janssen by Johnson & Johnson) was administered, the frequency, length, and quantity of the menstrual cycle after the administration, and how long this effect lasted in case of menstrual cycle irregularities and/or abnormal uterine bleeding.

In women of reproductive age who were not using hormonal therapies, any frequency shorter than 25 days or longer than 36 days, any length shorter than 3 days or longer than 7 days, and any quantity estimated less or more than previous cycles was defined as abnormal.

We considered days 1–3 of the cycle as menstruation, days 4–9 as the early-mid follicular phase, days 10–14 as the late follicular phase, days 14–21 as the early-mid luteal phase, and days 21–28 as the late luteal phase.

The questionnaire was designed to be self-administered, only one time per respondent, and without any restriction regarding the timing of the first or second dose of the vaccine. The survey was available only in the Italian language, for 30 days, (10 September 2021; 10 October 2021) and distributed by social media (LinkedIn, Facebook, and Twitter).

To limit confounders, in the current analysis, we excluded women with gynaecological and non-gynaecological diseases, undergoing hormonal and non-hormonal treatments, in perimenopause or menopause, as well as who had irregular menstrual cycle in the last 12 months before vaccine administration.

### 2.2 Study registration and ethical and methodological standards

The study was registered on ClinicalTrial.gov (registration ID: NCT05083065) before starting the enrolment. The design, analysis, interpretation of data, drafting, and revisions conform to the Helsinki Declaration, the Committeeon Publication Ethics (COPE) guidelines (http://publicationethics.org/), and the Checklist for Reporting Results of Internet E-surveys [12], available through the enhancing the quality and transparency of health research (EQUATOR) network (www.equator-network.org). The data collected through the survey were anonymised, taking into account the observational nature of the study, without personal data that could lead to formal identification, so a formal Institutional Review Board (IRB) approval was not mandatory (Code of Federal Regulation, Title 45, part 46, subpart A, sec. 46.101, available through the Office for Human Research Protections, Rockville, USA, and validated by the American Association for Public Opinion Research, Washington, USA). Each patient enrolled in this study gave informed consent to allow data collection and analysis for research purposes before starting the survey. The study was not supported by any fund/grant, and no remuneration was offered to encourage patients to give consent to enter, continue, or complete the survey.

### 2.3 Statistical analysis

Statistical analyses were mainly descriptive. We reported absolute and relative frequencies for the frequency, length, and quantity of the menstrual cycle after vaccine administration. For selected bivariate associations (frequency by the vaccine type; and frequency, length, and quantity of the menstrual cycle after vaccine administration by the menstrual phase), we reported the *p*-values from chi-square tests. The cutpoint for the level of significance was set as *p* < 0.05. For the statistical analyses, we used SAS software, 9.4 release.


**Informed consent:** Informed consent has been obtained from all individuals included in this study.
**Ethical approval:** Formal IRB approval for this survey was not mandatory (Code of Federal Regulation, Title 45, part 46, subpart A, sec. 46.101, available through the Office for Human Research Protections, Rockville, USA, and validated by the American Association for Public Opinion Research, Washington, USA).

## 3 Results

From 10 September to 10 October 2021, 369 women answered the online questionnaire. We excluded 205 women due to one or more of the following criteria: women with gynaecological and non-gynaecological diseases, undergoing hormonal and non-hormonal treatments, in perimenopause or menopause, as well as who had irregular menstrual cycle in the last 12 months before the vaccine administration. Among the remaining 164 women, who were in reproductive age and declared regular menstrual cycle before the vaccine administration, mean age was 35.8 ± 7.2 years, mean weight was 62.0 ± 14.3 kg, and mean height was 164.5 ± 6.3 cm (mean BMI: 22.9 ± 5.0).

### 3.1 Menstrual cycle analysis after the first dose of vaccine

Among the whole population who received at least the first dose (single in the case of Janssen by Johnson & Johnson), 9 women had the first dose of vaccine using Vaxzevria (AstraZeneca), 133 using Comirnaty (Pfizer-BioNTech), 19 using Spikevax (Moderna), and 3 using Janssen (Johnson & Johnson).

The first dose of vaccine was administered during menstruation in 23 women, during the early-mid follicular phase in 26 women, during the late follicular phase in 36 women, during the early-mid luteal phase in 19 women, and during the late luteal phase in 19 women; 41 women declared they did not remember the phase of the menstrual cycle during which the first dose of the vaccine was administered.

The analysis of frequency, length, and quantity of the menstrual cycle after the administration of the first dose of the vaccine is reported in Table 1, stratified for the type of vaccine and the phase of the menstrual cycle during which the vaccine was administered.

**Table 1:** Analysis of frequency, length, and quantity of the menstrual cycle after the administration of the first dose of vaccine, stratified for the type of vaccine and the phase of the menstrual cycle during which the vaccine was administered

	Type of vaccine				Phase of the menstrual cycle during administration					
	Vaxzevria (AstraZeneca)	Comirnaty (Pfizer-BioNtech)	Spikevax (Moderna)	Janssen (Johnson & Johnson)	Menstruation	Early-mid follicular phase	Late follicular phase	Early-mid luteal phase	Late luteal phase	Unknown
**Frequency**										
I did not notice any variation of the frequency	3 (33.3%)	58 (42.9%)	10 (52.6%)	2 (66.7%)	11 (43.5%)	9 (34.6%)	13 (36.1%)	4 (21.1%)	9 (47.4%)	27 (65.9%)
I did not have menstrual cycle	1 (11.1%)	6 (4.5%)	1 (5.3%)	0	1 (4.3%)	1 (3.8%)	0	3 (15.8%)	1 (5.3%)	2 (4.9%)
Menstruation arrived 1–5 days earlier than expected	3 (33.3%)	24 (18%)	4 (21.1%)	1 (33.3%)	5 (21.7%)	7 (26.9%)	7 (19.4%)	3 (15.8%)	4 (21.1%)	6 (14.6%)
Menstruation arrived 5–10 days earlier than expected	1 (11.1%)	9 (6.8%)	2 (10.5%)	0	1 (4.3%)	2 (7.7%)	3 (8.3%)	3 (15.8%)	1 (5.3%)	2 (4.9%)
Menstruation arrived more than 10 days earlier than expected	1 (11.1%)	11 (8.3%)	1 (5.3%)	0	2 (8.7%)	4 (15.4%)	4 (11.1%)	1 (5.3%)	1 (5.3%)	1 (2.4%)
Menstruation arrived 1–5 days later than expected	0	12 (9%)	1 (5.3%)	0	0	2 (7.7%)	4 (11.1%)	3 (15.8%)	3 (15.8%)	1 (2.4%)
Menstruation arrived 5–10 days later than expected	0	7 (5.3%)	0	0	1 (4.3%)	0	4 (11.1%)	2 (10.5%)	0	0
Menstruation arrived more than 10 days later than expected	0	6 (4.3%)	0	0	2 (8.7%)	1 (3.8%)	1 (2.8%)	0	0	2 (4.9%)
Totals	9 (100%)	133 (100%)	19 (100%)	3 (100%)	23 (100%)	26 (100%)	36 (100%)	19 (100%)	19 (100%)	41 (100%)
**Length**										
I did not notice any variation of the length	4 (44.4%)	78 (58.6%)	9 (47.4%)	1 (33.3%)	13 (56.5%)	15 (57.7%)	21 (58.3%)	6 (31.6%)	11 (57.9%)	26 (63.4%)
I had spotting	2 (22.2%)	12 (9%)	0	0	2 (8.7%)	2 (7.7%)	2 (5.6%)	3 (15.8%)	1 (5.3%)	4 (9.8%)
Menstruation lasted more than 7 days	2 (22.2%)	21 (15.8%)	3 (15.8%)	2 (66.7%)	4 (17.4%)	5 (19.2%)	6 (16.7%)	7 (36.8%)	3 (15.8%)	3 (7.3%)
Menstruation lasted less than 3 days	1 (11.1%)	22 (16.5%)	7 (36.8%)	0	4 (17.4%)	4 (15.4%)	7 (19.4%)	3 (15.8%)	4 (21.1%)	8 (19.5%)
Totals	9 (100%)	133 (100%)	19 (100%)	3 (100%)	23 (100%)	26 (100%)	36 (100%)	19 (100%)	19 (100%)	41 (100%)
**Quantity**										
I did not notice any variation of the quantity	3 (33.3%)	71 (52.6%)	9 (47.4%)	1 (33.3%)	12 (52.2%)	12 (46.2%)	20 (55.6%)	5 (26.3%)	10 (52.6%)	25 (58.5%)
Menstruation was heavier than usual	5 (55.6%)	33 (24.8%)	3 (15.8%)	2 (66.7%)	6 (26.1%)	11 (42.3%)	8 (22.2%)	11 (57.9%)	3 (15.8%)	4 (9.8%)
Menstruation was less heavy than usual	1 (11.1%)	29 (21.8%)	7 (36.8%)	0	5 (21.7%)	3 (11.5%)	8 (22.2%)	3 (15.8%)	6 (31.6%)	12 (29.3%)
Totals	9 (100%)	133 (100%)	19 (100%)	3 (100%)	23 (100%)	26 (100%)	36 (100%)	19 (100%)	19 (100%)	41 (100%)

Among the women who had menstrual cycle irregularities after the first dose (*n* = 94), these occurred only during the first month after the vaccination in 28 (29.8%) cases, only during the second month after the vaccination in 5 (5.3%) cases, lasted for both the first and second month in 19 (20.2%) cases, and finally was reported for more than two months in 42 (44.7%) cases.

### 3.2 Menstrual cycle analysis after the second dose of vaccine

Among the 135 women who received also the second vaccine dose, 8 women had the second dose of vaccine using Vaxzevria (AstraZeneca), 113 using Comirnaty (Pfizer-BioNTech), 14 using Spikevax (Moderna).

The second dose of vaccine was administered during menstruation in 24 women, during the early-mid follicular phase in 22 women, during the late follicular phase in 19 women, during the early-mid luteal phase in 14 women, and during the late luteal phase in 17 women; 39 women declared they did not remember the phase of the menstrual cycle during which the first dose of the vaccine was administered.

The analysis of frequency, length, and quantity of the menstrual cycle after the administration of the second vaccine dose is reported in Table 2, stratified for the type of vaccine and the phase of the menstrual cycle during which the vaccine was administered.

**Table 2:** Analysis of frequency, length, and quantity of the menstrual cycle after the administration of the second dose of the vaccine, stratified for the type of vaccine and the phase of the menstrual cycle during which the vaccine was administered

	Type of vaccine			Phase of the menstrual cycle during administration					
	Vaxzevria (AstraZeneca)	Comirnaty (Pfizer-BioNtech)	Spikevax (Moderna)	Menstruation	Early-mid follicular phase	Late follicular phase	Early-mid luteal phase	Late luteal phase	Unknown
**Frequency**									
I did not notice any variation of the frequency	2 (25%)	54 (47.8%)	3 (21.4%)	9 (37.5%)	13 (59.1%)	6 (31.6%)	8 (57.1%)	4 (23.5%)	19 (48.7%)
I did not have menstrual cycle	0	6 (5.3%)	1 (7.1%)	1 (4.2%)	0	1 (5.3%)	1 (7.1%)	2 (11.8%)	2 (5.1%)
Menstruation arrived 1–5 days earlier than expected	3 (37.5%)	14 (12.4%)	5 (35.7%)	4 (16.7%)	1 (4.5%)	2 (10.5%)	3 (21.4%)	6 (35.3%)	6 (15.4%)
Menstruation arrived 5–10 days earlier than expected	1 (12.5%)	11 (9.7%)	2 (14.3%)	3 (12.5%)	2 (9.1%)	4 (21.1%)	0	3 (17.6%)	2 (5.1%)
Menstruation arrived more than 10 days earlier than expected	1 (12.5%)	8 (7.1%)	2 (14.3%)	2 (8.3%)	3 (13.6%)	4 (21.1%)	1 (7.1%)	0	1 (2.6%)
Menstruation arrived 1–5 days later than expected	0	8 (7.1%)	1 (7.1%)	3 (12.5%)	1 (4.5%)	0	0	0	5 (12.8%)
Menstruation arrived 5–10 days later than expected	1 (12.5%)	3 (2.7%)	0	1 (4.2%)	1 (4.5%)	1 (5.3%)	0	1 (5.9%)	0
Menstruation arrived more than 10 days later than expected	0	9 (8%)	0	1 (4.2%)	1 (4.5%)	1 (5.3%)	1 (7.1%)	1 (5.9%)	4 (10.3%)
Totals	8 (100%)	113 (100%)	14 (100%)	24 (100%)	22 (100%)	19 (100%)	14 (100%)	17 (100%)	39 (100%)
**Length**									
I did not notice any variation of the length	4 (50%)	65 (57.5%)	8 (57.1%)	15 (62.5%)	13 (59.1%)	6 (31.6%)	7 (50%)	11 (64.7%)	25 (64.1%)
I had spotting	0	5 (4.4%)	0	0	0	0	0	0	5 (12.8%)
Menstruation lasted more than 7 days	4 (50%)	24 (21.2%)	4 (28.6%)	5 (20.8%)	6 (27.3%)	9 (47.4%)	5 (35.7%)	4 (23.5%)	3 (7.7%)
Menstruation lasted less than 3 days	0	19 (16.8%)	2 (14.3%)	4 (16.7%)	3 (13.6%)	4 (21.1%)	2 (14.3%)	2 (11.8%)	6 (15.4%)
Totals	8 (100%)	113 (100%)	14 (100%)	24 (100%)	22 (100%)	19 (100%)	14 (100%)	17 (100%)	39 (100%)
**Quantity**									
I did not notice any variation of the quantity	3 (37.5%)	61 (53.1%)	6 (35.7%)	10 (41.7%)	15 (63.6%)	9 (42.1%)	7 (50%)	6 (35.3%)	23 (59%)
Menstruation was heavier than usual	5 (62.5%)	32 (28.3%)	4 (28.6%)	9 (37.5%)	4 (18.2%)	7 (36.8%)	6 (42.9%)	7 (41.2%)	8 (20.5%)
Menstruation was less heavy than usual	0	20 (17.7%)	4 (28.6%)	5 (20.8%)	3 (13.6%)	3 (15.8%)	1 (7.1%)	4 (23.5%)	8 (20.5%)
Totals	8 (100%)	113 (100%)	14 (100%)	24 (100%)	22 (100%)	19 (100%)	14 (100%)	17 (100%)	39 (100%)

Among the women who had menstrual cycle irregularities after the second dose (*n* = 84), these occurred only during the first month after the vaccination in 19 (22.6%) cases, only during the second month after the vaccination in 6 (7.1%) cases, lasted for both the first and second month in 21 (25%) cases, and finally was reported for more than two months in 38 (45.2%) cases.

## 4 Discussion

### 4.1 Main findings after the first dose of vaccine

After the first dose of vaccine, 66.7% of women who received Vaxzevria (AstraZeneca), 57.1% of women who received Comirnaty (Pfizer-BioNTech), 47.4% of women who received Spikevax (Moderna), and 33.3% of women who received Janssen (Johnson & Johnson) reported alterations in the frequency of the subsequent menstrual cycle. Most of these women had menstruation 1–5 days earlier than expected, and this alteration occurred mainly when the first dose of vaccine was administered during the first 14 days of the menstrual cycle. However, we did not find significant differences (*p* = 0.45) in the occurrence of this menstrual irregularity based on when the first vaccine dose was administered (follicular versus luteal phase). Moreover, we did not find significant differences (*p* = 0.60) between recombinant (Vaxzevria and Janssen) and mRNA vaccines (Comirnaty and Moderna).

Additionally, after the first dose, 55.6% of women who received Vaxzevria (AstraZeneca), 41.4% of women who received Comirnaty (Pfizer-BioNTech), 52.6% of women who received Spikevax (Moderna), and 66.7% of women who received Janssen (Johnson & Johnson) reported alteration in the length of the subsequent menstrual cycle. Among these women, the most common alteration was menstruation that lasted more than usual, which occurred mainly when the vaccine was administered during the early luteal phase, although we did not find significant differences (*p* = 0.90) based on when the first vaccine dose was administered (follicular versus luteal phase).

Finally, after the first dose, 66.7% of women who received Vaxzevria (AstraZeneca), 47.4% of women who received Comirnaty (Pfizer-BioNTech), 52.6% of women who received Spikevax (Moderna), and 66.7% of the women who received Janssen (Johnson & Johnson) declared alteration in the quantity of the subsequent menstrual flow. Among these women, the most common alteration was heavier menstruation than usual, which occurred mainly when the vaccine was administered during the first 14 days of the menstrual cycle. We did not find significant differences (*p* = 0.77) based on when the first vaccine dose was administered (follicular versus luteal phase). Overall, menstrual cycle irregularities after the first dose of the vaccine spontaneously resolved in approximately half the cases within two months.

### 4.2 Main findings after the second dose of vaccine

After the second dose of vaccine, 75% of women who received Vaxzevria (AstraZeneca), 52.2% of women who received Comirnaty (Pfizer-BioNTech), and 78.6% of women who received Spikevax (Moderna) reported alteration in the frequency of the subsequent menstrual cycle(s). Similar to the first dose, the most common frequency alteration was menstruation that arrived 1–5 days earlier than expected. We did not find significant differences (*p* = 0.51) between recombinant (Vaxzevria and Janssen) and mRNA vaccines (Comirnaty and Moderna) or based on when the second vaccine dose was administered (follicular versus luteal phase; *p* = 0.11).

In addition, 50% of women who received Vaxzevria (AstraZeneca), 42.5% of the women who received Comirnaty (Pfizer-BioNTech), and 42.9% of the women who received Spikevax (Moderna) declared alteration in the length of the subsequent menstrual cycle after the second dose. Consistently with the first dose, the most common occurrence was menstruation that lasted more than usual. We did not find significant differences (*p* = 0.67) based on when the second vaccine dose was administered (follicular versus luteal phase).

Finally, 62.5% of women who received Vaxzevria (AstraZeneca), 46.9% of women who received Comirnaty (Pfizer-BioNTech), and 64.3% of women who received Spikevax (Moderna) declared alteration in the quantity in the subsequent menstrual flow after the second dose, and the most common occurrence was menstruation heavier than usual. We did not find significant differences (*p* = 0.69) based on when the second vaccine dose was administered (follicular versus luteal phase). Similar to the first dose, menstrual cycle irregularities after the second dose of vaccine spontaneously resolved in approximately half of the cases within two months.

### 4.3 Strengths and limitations

To the best of our knowledge, this is one of the few preliminary reports aimed to investigate menstrual cycle irregularities among women who received the first and second doses of the COVID-19 vaccine. To avoid potential biases, we excluded from the current analysis women with gynaecological and non-gynaecological diseases, undergoing hormonal and non-hormonal treatments, in perimenopause or menopause, as well as who had irregular menstrual cycle in the last 12 months before the vaccine administration. In addition, we investigated the alteration of frequency, length, and quantity of the subsequent menstrual cycles, stratifying data for the type of vaccine and the phase of the menstrual cycle during which the first and second doses were administered.

Nevertheless, several limitations should be taken into account for a proper and cautious data interpretation: first of all, we used a customised questionnaire, without a previous validation; second, this questionnaire was self-administered and diffused among the general population through social media, so we cannot rule out that women who had menstrual cycle irregularities after COVID-19 vaccination were more motivated to answer than women who did not experience this event; for the same reason, we could not exactly measure the quantity of the menstrual cycle, and we left this parameter as a subjective evaluation from the patient’s perspective (more or less heavy than usual), whereas we used exact ranges for the frequency and length of the menstrual cycle. Overall, the recall bias may be significant for this preliminary report. From the methodological point of view, we do not have a control group, so our report is aimed only to offer a description of what we observed, without any possibility to infer cause–effect. Finally, the number of women who answered the questionnaire is limited, impeding the observation of statistically significant associations between menstrual irregularities and the vaccine type or the menstrual phase at the time of administration.

### 4.4 Interpretation and comparison with other literature studies

Overall, our preliminary report highlights that approximately 50–60% of reproductive age women who received the first dose of COVID-19 vaccine had menstrual cycle irregularities, regardless of the type of vaccine administered. The occurrence of menstrual irregularities seems to be slightly higher (60–70%) after the second dose, suggesting a potential additive effect. After both the first and second doses of vaccine, the most common alterations seem to be anticipated, longer, and heavier menstrual cycle than expected and usual.

Currently, more than 30,000 reports of irregularities in the menstrual cycle have been reported by 2 September 2021, across all COVID-19 vaccines [13]. A longitudinal study to explore the potential impacts of COVID-19 vaccination on menstruation is currently ongoing, supported by the NIH’s Eunice Kennedy Shriver National Institute of Child Health and Human Development and the NIH Office of Research on Women’s Health [9]. Although the regulatory agencies did not find convincing evidence of a link between changes to menstrual periods and COVID-19 vaccines, concern regarding this specific issue has been raised by an editorial recently published in the British Journal of Medicine [8]. According to recent data comparing women who received vaccination and unvaccinated controls [14], COVID-19 vaccine was associated with a less than 1 day change in the cycle length for both vaccine-dose cycles compared with pre-vaccine cycles (first dose 0.71 day-increase, 98.75% confidence interval (CI) 0.47–0.94; second dose 0.91, 98.75% CI 0.63–1.19). Other reports, still in the pre-print phase, have provided further support to the hypothesis. Menstrual abnormalities have been recorded in 20–42% of vaccinated women, while 66% of post-menopausal women reported breakthrough bleeding [15,16]. Our results are in line with these last reports.

Menstrual changes following vaccination are indeed not so unusual, given that such modifications were observed after vaccination for other microbes, like the human papilloma virus [17], or human hormones, such as human chorionic gonadotropin [18]. Such disturbances could likely be ascribed to the inflammatory/immunological reaction ensuing from adjuvants comprised in the vaccines, at least in some cases [19]. However, we cannot discard that the COVID-19-related spike protein could exert a causative pathogenic role, as similar changes in menstrual cycles have been recorded during COVID-19 infection [20]. Moreover, diffusion of the spike protein in women tissues – either linked to COVID-19 infection or released after mRNA-based vaccination – can also interfere with the endocrine homeostasis of the menstrual cycle, given that the use of combined oral contraceptives was associated with lower odds of reporting any menstrual changes [15]. These findings provide further confirmation of the protective effect of oestrogens in mitigating the severity of COVID-19-related clinical outcomes, as previously reported [21].

Although speculative, we may hypothesise that what we observed could be due, at least in part, to phase-specific hormonal variation caused by potential pro-inflammatory and pro-coagulative changes. Indeed, several pieces of evidence suggest crosstalk between inflammatory homeostasis and menstrual cycle regulation [10], which may be slightly disturbed by temporary hormonal variation and secondary to the inflammatory reaction induced by the vaccine. Remarkably, reproductive toxicity studies performed in the mouse models with Comirnaty (Pfizer-BioNTech) [22], Spikevax (Moderna) [23], Vaxzevria (AstraZeneca) [24], and Janssen (Johnson & Johnson) [25] reveal no special hazard for humans regarding fertility. Although reported changes to the menstrual cycle after vaccination are temporary and self-limiting and no cases resulted in clinically significant consequences, the link between COVID-19 vaccination and irregularities in the menstrual cycle deserves to be investigated in further specific studies. We hope that the results of our study will help for a proper counselling and mitigate fear about potential side effects of COVID-19 vaccination, even considering the potential detrimental role of COVID-19 pandemic itself on the quality of life and mental health [26,27].

## 5 Conclusion

According to our preliminary report, more than half of reproductive-age women who received the first and second doses of COVID-19 vaccine had menstrual cycle irregularities at least in the following menstrual cycle, regardless of the vaccine type and the phase of the menstrual cycle during which the vaccine was administered. However, the occurrence of menstrual irregularities after both the first and second doses of the vaccine was found to self-resolve in approximately half the cases within two months, without clinically relevant consequences.

Although we solicit further studies to confirm or disregard this observational evidence on large dataset analysis, we suggest considering this element during the counselling of women who received the COVID-19 vaccine, letting them know about the potential occurrence of a temporary and self-limiting menstrual cycle irregularity in the subsequent month(s). In addition, we take the opportunity to highlight clearly that our preliminary report does not allow us to draw any firm conclusion about a potential cause–effect correlation between the COVID-19 vaccine and menstrual irregularities, or about any potential fertility impairment.

## References

[1] Palmieri L, Palmer K, Lo Noce C, Meli P, Giuliano M, Floridia M, et al. Italian national institute of health COVID-19 mortality group (*). Differences in the clinical characteristics of COVID-19 patients who died in hospital during different phases of the pandemic: national data from Italy. Aging Clin Exp Res. 2021;33(1):193–9. 10.1007/s40520-020-01764-0. PMC7750107 33345291

[2] Hui DS, Azhar E, Madani TA, Ntoumi F, Kock R, Dar O, et al. The continuing 2019-nCoV epidemic threat of novel coronaviruses to global health – the latest 2019 novel coronavirus outbreak in Wuhan, China. Int J Infect Dis. 2020;91:264–6. 10.1016/j.ijid.2020.01.009. PMC7128332 31953166

[3] Perico L, Tomasoni S, Peracchi T, Perna A, Pezzotta A, Remuzzi G, et al. COVID-19 and lombardy: TESTing the impact of the first wave of the pandemic. EBioMedicine. 2020;61:103069. 10.1016/j.ebiom.2020.103069. PMC7581396 33130396

[4] Alanezi F, Aljahdali A, Alyousef SM, Alrashed H, Mushcab H, AlThani B, et al. A comparative study on the strategies adopted by the United Kingdom, India, China, Italy, and Saudi Arabia to contain the spread of the COVID-19 pandemic. J Healthc Leadersh. 2020;12:117–31. 10.2147/JHL.S266491. PMC7608628 33154693

[5] Roncati L, Roncati M. COVID-19 “Green Pass”: a lesson on the proportionality principle from galicia. Eur J Health Law. 2021. 10.1163/15718093-bja10055. 34706331

[6] Kamin-Friedman S, Peled Raz M. Lessons from Israel’s COVID-19 green pass program. Isr J Health Policy Res. 2021;10(1):61. 10.1186/s13584-021-00496-4. PMC8554179 34715931

[7] Cari L, Alhosseini MN, Fiore P, Pierno S, Pacor S, Bergamo A, et al. Cardiovascular, neurological, and pulmonary events following vaccination with the BNT162b2, ChAdOx1 nCoV-19, and Ad26.COV2.S vaccines: An analysis of European data. J Autoimmun. 2021;125:102742. 10.1016/j.jaut.2021.102742. PMC8547775 34710832

[8] Male V. Menstrual changes after covid-19 vaccination. BMJ. 2021;374:n2211. 10.1136/bmj.n2211. 34526310

[9] Eunice Kennedy Shriver National Institute of Child Health and Human Development. Item of interest: NIH funds studies to assess potential effects of COVID-19 vaccination on menstruation. [Internet]; 2021. [cited 2021 Dec 27]. https://www.nichd.nih.gov/newsroom/news/083021-COVID-19-vaccination-menstruation.

[10] Li K, Chen G, Hou H, Liao Q, Chen J, Bai H, et al. Analysis of sex hormones and menstruation in COVID-19 women of child-bearing age. Reprod Biomed Online. 2021;42(1):260–7. 10.1016/j.rbmo.2020.09.020. PMC7522626 33288478

[11] Demir O, Sal H, Comba C. Triangle of COVID, anxiety and menstrual cycle. J Obstet Gynaecol. 2021;41(8):1257–61. 10.1080/01443615.2021.1907562. 33955327

[12] Eysenbach G. Improving the quality of Web surveys: the Checklist for Reporting Results of Internet E-Surveys (CHERRIES). J Med Internet Res. 2004;6(3):e34. 10.2196/jmir.6.3.e34, Erratum in: 10.2196/jmir.2042. PMC1550605 15471760

[13] Medicines and Healthcare Products Regulatory Agency. Coronavirus vaccine – weekly summary of yellow card reporting. [Internet]; 2021. [cited 2021 Dec 27]. https://www.gov.uk/government/publications/coronavirus-covid-19-vaccine-adverse-reactions/coronavirus-vaccine-summary-of-yellow-card-reporting.

[14] Edelman A, Boniface ER, Benhar E, Han L, Matteson KA, Favaro C, et al. Association between menstrual cycle length and coronavirus disease 2019 (COVID-19) vaccination: a U.S. Cohort. Obstet Gynecol. 2022. 10.1097/AOG.0000000000004695. PMC8936155 34991109

[15] Lee KMN, Junkins EJ, Fatima UA, Cox ML, Clancy KBH. Characterizing menstrual bleeding changes occurring after SARS-CoV-2 vaccination. [Preprint]; 2021. [cited 2021 Dec 27]. 10.1101/2021.10.11.21264863.

[16] Alvergne A, Kountourides G, Argentieri MA, Agyen L, Rogers N, Knight D, et al. COVID-19 vaccination and menstrual cycle changes: A 2 United Kingdom (UK) retrospective case-control study. [Preprint]; 2021. [cited 2021 Dec 27]. 10.1101/2021.11.23.21266709. PMC10015085 36987520

[17] Suzuki S, Hosono A. No association between HPV vaccine and reported post-vaccination symptoms in Japanese young women: Results of the Nagoya study. Papillomavirus Res. 2018;5:96–103. 10.1016/j.pvr.2018.02.002. PMC5887012 29481964

[18] Kharat I, Nair NS, Dhall K, Sawhney H, Krishna U, Shahani SM, et al. Analysis of menstrual records of women immunized with anti-hCG vaccines inducing antibodies partially cross-reactive with hLH. Contraception. 1990;41(3):293–9. 10.1016/0010-7824(90)90070-c. 2182289

[19] Colafrancesco S, Perricone C, Tomljenovic L, Shoenfeld Y. Human papilloma virus vaccine and primary ovarian failure: another facet of the autoimmune/inflammatory syndrome induced by adjuvants. Am J Reprod Immunol. 2013;70(4):309–16. 10.1111/aji.12151. 23902317

[20] Costeira R, Lee KA, Murray B, Christiansen C, Castillo-Fernandez J, Ni Lochlainn M, et al. Estrogen and COVID-19 symptoms: associations in women from the COVID Symptom Study. PLoS One. 2021;16(9):e0257051. 10.1371/journal.pone.0257051. PMC8432854 34506535

[21] Critchley HOD, Maybin JA, Armstrong GM, Williams ARW. Physiology of the endometrium and regulation of menstruation. Physiol Rev. 2020;100(3):1149–79. 10.1152/physrev.00031.2019. 32031903

[22] EMA/707383/2020 Corr.1*1 (19 February 2021) Committee for Medicinal Products for Human Use (CHMP). Assessment report – Comirnaty – Common name: COVID-19 mRNA vaccine (nucleoside-modified). Procedure No. EMEA/H/C/005735/0000. [Internet]; 2021. [cited 2021 Dec 27]. https://www.ema.europa.eu/en/documents/assessment-report/comirnaty-epar-public-assessment-report_en.pdf.

[23] EMA/15689/2021 Corr.1*1 (11 March 2021) Committee for Medicinal Products for Human Use (CHMP). Assessment report – COVID-19 Vaccine Moderna – Common name: COVID-19 mRNA Vaccine (nucleoside-modified). Procedure No. EMEA/H/C/005791/0000.[Internet]; 2021. [cited 2021 Dec 27]. https://www.ema.europa.eu/en/documents/assessment-report/spikevax-previously-covid-19-vaccine-moderna-epar-public-assessment-report_en.pdf.

[24] EMA/94907/2021 (29 January 2021) Committee for Medicinal Products for Human Use (CHMP). Assessment report – COVID-19 Vaccine AstraZeneca – Common name: COVID-19 Vaccine (ChAdOx1-S [recombinant]). Procedure No. EMEA/H/C/005675/0000. [Internet]; 2021. [cited 2021 Dec 27]. https://www.ema.europa.eu/en/documents/assessment-report/vaxzevria-previously-covid-19-vaccine-astrazeneca-epar-public-assessment-report_en.pdf.

[25] EMA/158424/2021 (11 March 2021) Committee for Medicinal Products for Human Use (CHMP). Assessment report – COVID-19 Vaccine Janssen – Procedure No. EMEA/H/C/005737/0000. [Internet]; 2021. [cited 2021 Dec 27]. https://www.ema.europa.eu/en/documents/assessment-report/covid-19-vaccine-janssen-epar-public-assessment-report_en.pdf.

[26] Biviá-Roig G, La Rosa VL, Gómez-Tébar M, Serrano-Raya L, Amer-Cuenca JJ, Caruso S, et al. Analysis of the impact of the confinement resulting from COVID-19 on the lifestyle and psychological wellbeing of Spanish pregnant women: an internet-based cross-sectional survey. Int J Environ Res Public Health. 2020;17(16):5933. 10.3390/ijerph17165933. PMC7460363 32824191

[27] Kolker S, Biringer A, Bytautas J, Blumenfeld H, Kukan S, Carroll JC. Pregnant during the COVID-19 pandemic: an exploration of patients’ lived experiences. BMC Pregnancy Childbirth. 2021;21(1):851. 10.1186/s12884-021-04337-9. PMC8718994 34972506

